# Nail-patella syndrome: Lesser known but more sensitive dermatologic features than triangular lunulae

**DOI:** 10.1016/j.jdcr.2026.08.015

**Published:** 2026-08-15

**Authors:** Arman Haveric, Patricia Zhao, Aleksander Dedo, Sylvia Hsu

**Affiliations:** Department of Dermatology, Temple University Hospital, Philadelphia, Pennsylvania

**Keywords:** distal interphalangeal, fingernails, nail dystrophy, nail-patella syndrome, proximal interphalangeal

## Case presentation

A 20-year-old woman presented for treatment of acne vulgaris. On review of her medical records, it was noted that her previous dermatologist had diagnosed her with lichen planus of the nails and had prescribed halobetasol 0.05% ointment take medicine twice a day without any improvement. Examination of the patient’s upper extremities revealed bilateral cubitus valgus deformities and triangular lunulae. Her family history was significant in that her father and paternal grandfather had the same bone and nail findings, consistent with nail-patella syndrome (NPS).

**Question: In addition to triangular lunulae, which of the following features are seen in NPS? (Select all that apply).**

**A.** Absence of distal interphalangeal (DIP) joint skin folds
**B.** Clubbing
**C.** Hypoplastic thumbnails
**D.** Joint hypermobility
**E.** Pterygium

**Correct answers**: A, C, and D.

## Discussion

NPS is an autosomal dominant disorder characterized by a clinical tetrad of fingernail, knee, elbow, and iliac crest abnormalities.^1^ It arises secondary to loss-of-function mutations in the *LMX1B* gene on chromosome 9q34, which drives dorsal-ventral limb development, anterior eye development, and podocyte (cell in Bowman’s capsule in kidneys) differentiation.^2^ Accordingly, patients with NPS often present with abnormalities of limb development (including hypoplastic olecranons and patellae), frequently develop renal disease from glomerular basement membrane dysfunction, and are at increased risk of primary open-angle glaucoma and ocular hypertension.^1^ Its prevalence is reported as approximately 1/50,000.^1^^,^^2^

Dermatologic findings are by far the most common clinical feature of NPS, with over 96% of affected patients exhibiting nail abnormalities.^1^ The most well-known feature of the nails in NPS is triangular lunulae. However, several other features are less commonly known. These include:

• **Nail splitting.** Nails may be separated into 2 halves by a longitudinal cleft or ridge of skin.^1^^,^^3^
• **Absence of skin creases overlying the DIP joints.**^1^^,^^3^
• **Hypoplastic or absent thumbnails.** Nail hypoplasia, aplasia or dystrophy is common. The thumb is often the most severely affected digit, and severity of nail dystrophy typically decreases sequentially from the index to the fifth finger.^1^
• **Abnormalities in joint mobility.** Hyperextension of the proximal interphalangeal joints and hyperflexion of the DIP joints is often seen, often resulting in “swan neck” appearance of the finger.^1^ Fifth finger clinodactyly (either ulnar or radial deviation) may also be seen.^1^

**Fig 1:**
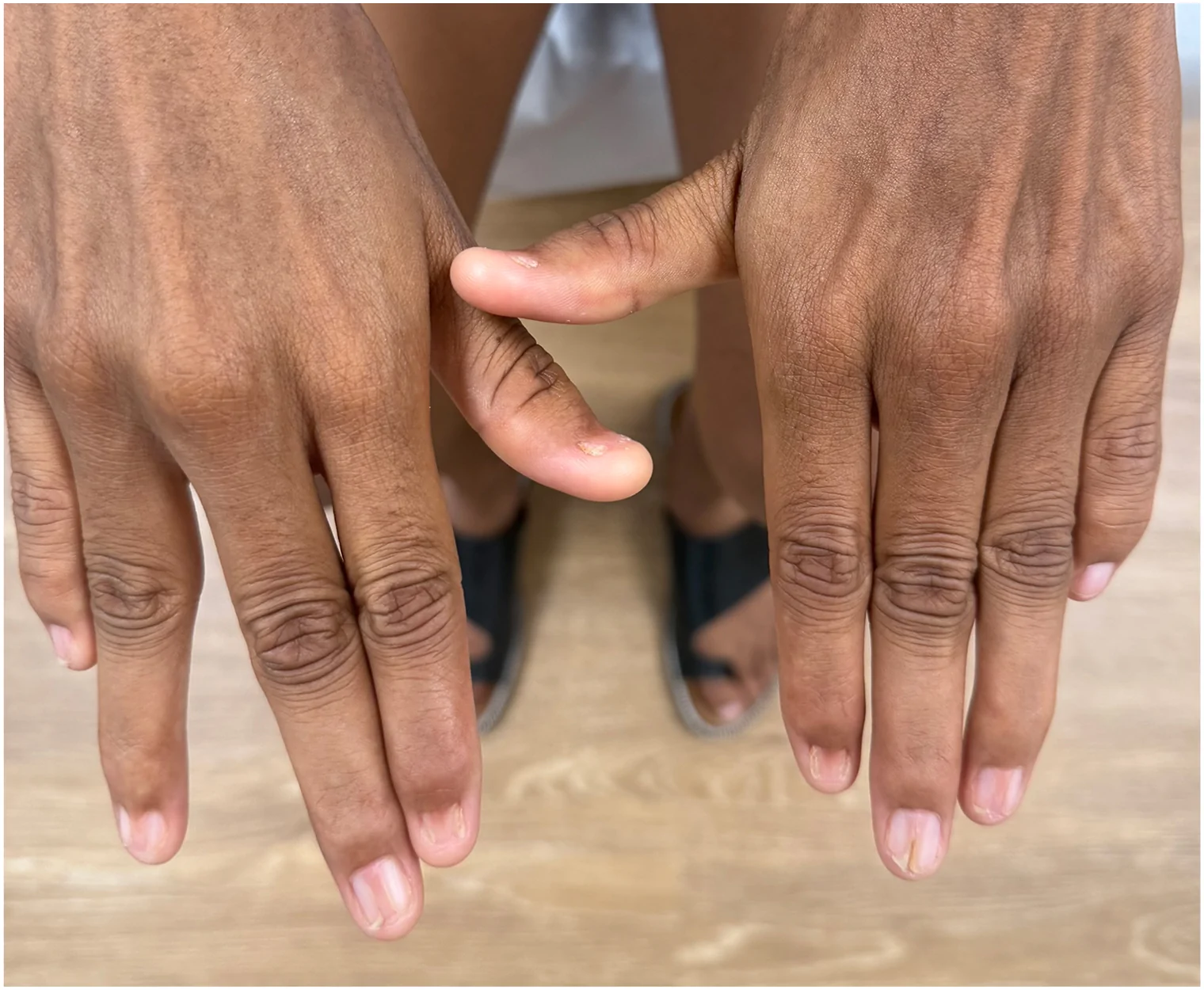
Fingernail splitting, absence of DIP skin folds, and fifth finger clinodactyly in NPS.

**Fig 2:**
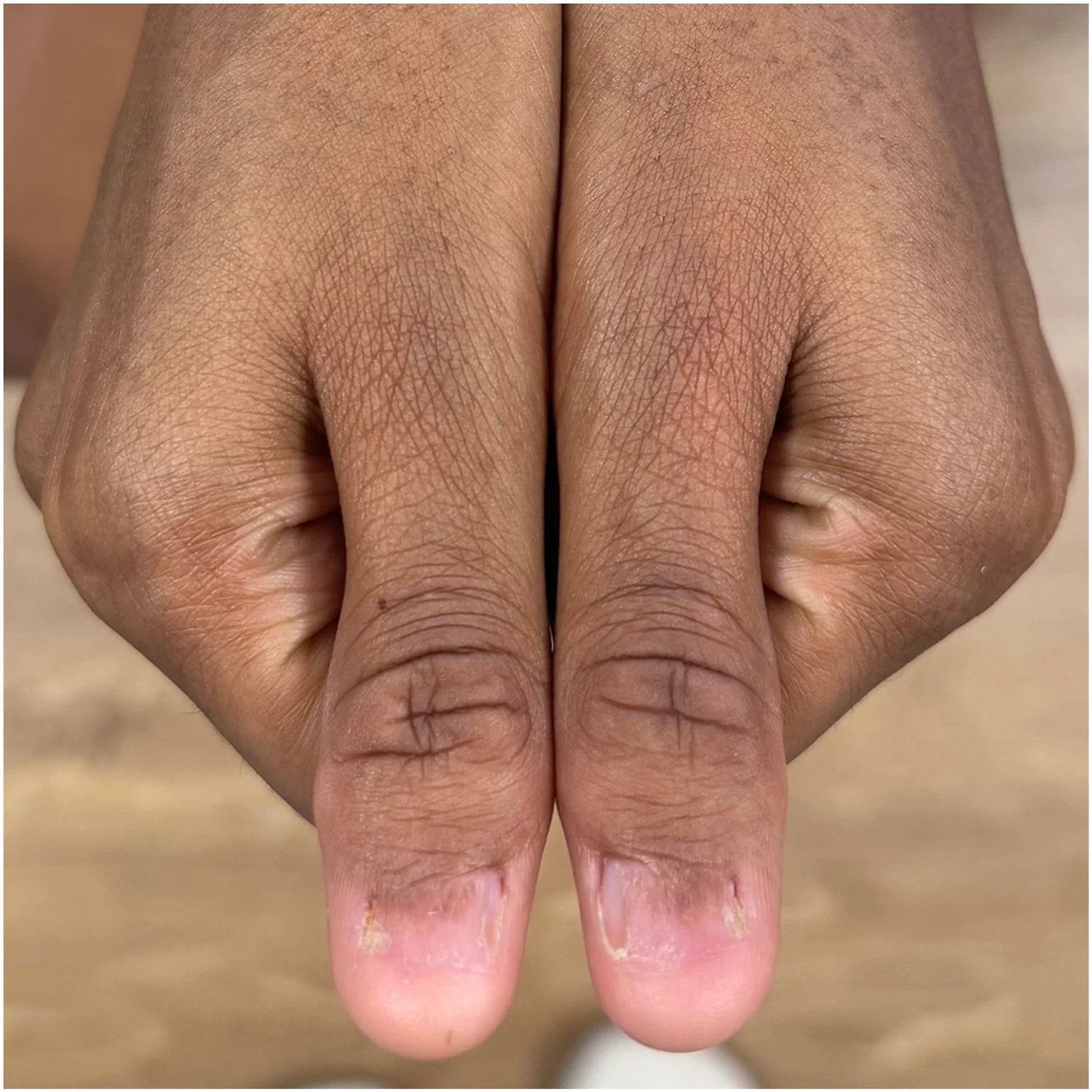
Thumbnail hypoplasia with increased dysplasia on the ulnar border of each thumbnail.

**Fig 3:**
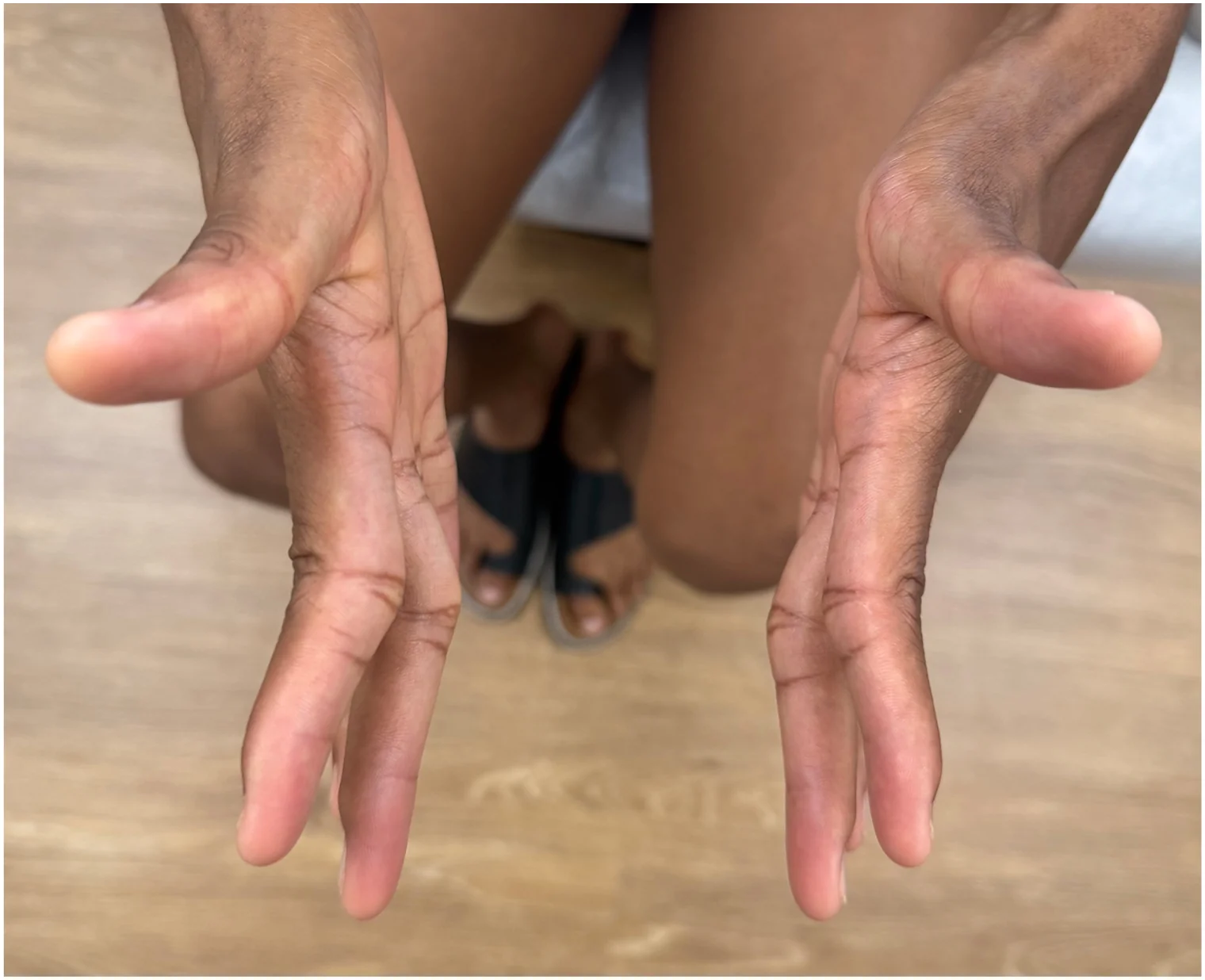
Swan-neck deformity due to flexion of the DIP joint and hyperextension of the proximal interphalangeal joint.

Our patient exhibited all of these features (Fig 1, Fig 2, Fig 3).

Although triangular lunulae are a near-universally recognized sign of NPS among dermatologists, other clinical features may be more sensitive for the disease. In a British study of 123 patients with NPS, loss of DIP skin creases was the most common finding, affecting 96% of subjects.^3^ Thumbnail dysplasia was seen in at least 68% of patients.^3^ There is some evidence that triangular lunulae may be less common in pediatric populations with NPS.^4^ Dermatologists are well-positioned to diagnose patients with nail-patella syndrome, and should be cognizant of the manifestations of the disease, as patients require multispecialty referral for long-term care.

Management of NPS primarily revolves around controlling orthopedic, renal, cardiovascular, and ophthalmologic sequelae of the disease. Patients frequently experience some degree of disability secondary to knee and hip dysplasia and should be referred to an orthopedic specialist for optimization of their functional capacity.^3^ Progression to end-stage renal disease can occur in as many as 15% of NPS patients and rates of ocular involvement in up to 25%.^1^^,^^3^

## Conflicts of interest

None disclosed.
